# Cardiovascular Fitness and Energy Expenditure Response during a Combined Aerobic and Circuit Weight Training Protocol

**DOI:** 10.1371/journal.pone.0164349

**Published:** 2016-11-10

**Authors:** Pedro J. Benito, María Alvarez-Sánchez, Víctor Díaz, Esther Morencos, Ana B. Peinado, Rocio Cupeiro, Nicola Maffulli, PRONAF Study Group

**Affiliations:** 1 Department of Health and Human Performance, Faculty of Physical Activity and Sport Sciences, Technical University of Madrid, Madrid, Spain; 2 Institute of Veterinary Physiology, University of Zurich, Zurich, Switzerland; 3 Zurich Center for Integrative Human Physiology (ZIHP), Zurich, Switzerland; 4 Francisco de Vitoria University, Exercise and Sport Sciences, Madrid, Spain; 5 University of Salerno School of Medicine and Surgery, Department of Musculoskeletal Disorders, Salerno, Italy; 6 Centre for Sport and Exercise Medicine, Queen Mary University of London, Mile End Hospital, 275 Bancroft Road, London E1 4DG, England, United Kingdom; Vanderbilt University, UNITED STATES

## Abstract

**Objectives:**

The present study describes the oxygen uptake and total energy expenditure (including both aerobic and anaerobic contribution) response during three different circuit weight training (CWT) protocols of equivalent duration composed of free weight exercises, machine exercises, and a combination of free weight exercises intercalating aerobic exercise.

**Design:**

Controlled, randomized crossover designs.

**Methods:**

Subjects completed in a randomized order three circuit weight training protocols of the same duration (3 sets of 8 exercises, 45min 15s) and intensity (70% of 15 repetitions maximum). The circuit protocols were composed of free weight exercises, machine exercises, or a combination of free weight exercises with aerobic exercise. Oxygen consumption and lactate concentration were measured throughout the circuit to estimate aerobic and anaerobic energy expenditure respectively.

**Results:**

Energy expenditure is higher in the combined exercise protocol (29.9±3.6 ml/kg/min), compared with Freeweight (24.2±2.8ml/kg/min) and Machine (20.4±2.9ml/kg/min). The combined exercise protocol produced the highest total energy expenditure but the lowest lactate concentration and perceived exertion. The anaerobic contribution to total energy expenditure was higher in the machine and free weight protocols compared with the combined exercise protocol (6.2%, 4.6% and 2.3% respectively).

**Conclusions:**

In the proposed protocols, the combined exercise protocol results in the highest oxygen consumption. Total energy expenditure is related to the type of exercise included in the circuit. Anaerobic contributions to total energy expenditure during circuit weight training may be modest, but lack of their estimation may underestimate total energy expenditure.

**Trial Registration:**

ClinicalTrials.gov NCT01116856

## Introduction

Cardiorespiratory fitness is a powerful predictor of future health in people of all ages and gender, regardless of pre-existing health conditions [1]. Consequently, aerobic training has been strongly recommended as an effective way of improving overall health [2]. Aerobic exercise has also been the main exercise choice when designing weight-loss programs. However, muscle strength is also a strong predictor of future health [3, 4].

Resistance training is therefore increasingly recommended for health promotion purposes, and for weight-loss programs. Current needs for further research include to design intervention studies to accurately compare the effects on different health outcomes and weight loss of aerobic exercise with those from resistance exercise, or a combination of both. However, for these comparisons to be accurate, it is necessary to design exercise programs that are equivalent in energy expenditure (EE); otherwise, different effects could just result from different EE. This is particularly challenging when comparing aerobic exercise such as running on a treadmill, with resistance training, since the latter may have an important anaerobic component which also contributes to total EE. This anaerobic contribution is usually ignored in EE estimations, leading to an overall underestimation of the actual EE of a given exercise

When resistance exercise, an anaerobic activity, is performed for more than 2 minutes, it produces lower EE compared to aerobic exercises [5]. However, there is controversy about the relative contribution of each energy production system [6, 7]. Isolated resistance exercises (bench press) produced an aerobic cost of 10.49 and 16.25 kcal/min when the exercise was performed at 40 and 70% of 1 repetition maximum (1 RM), respectively [8]. Other resistance exercises involving large muscle masses (parallel squats) produce an aerobic energy cost of 10.85 and 18.98 kcal/min at 40 and 70% of the 1 RM [8]. These values are significantly greater than previously suggested at 5.93 and 5.63 kcal/min at 65% and 75% of the 1 RM, respectively [9, 10]. In these studies, the weight load lifted was strongly and significantly associated with oxygen consumption (VO_2_). However, this relationship may be misleading for higher intensities of exercises, where mechanical work is associated with an increased production of energy by anaerobic means [11]. In fact, total EE is higher in bouts with a high number of repetitions (i.e. greater mechanical work), with the anaerobic contribution accounting for as much as 42% of the total EE [11]. Total EE during isolated resistance exercise can be significantly underestimated if the anaerobic contribution is not taken into consideration [8, 12].

In this context, the present study aimed to describe the oxygen uptake and total energy expenditure (including both aerobic and anaerobic contribution) response during three different CWT protocols of equivalent duration composed of: i) free weight exercises, ii) machine exercises, and iii) a combination of free weight exercises intercalating aerobic exercise.

## Methods

### Study design, participants and protocols

All procedures described in the present study were approved by the Human Research Review Committee of the University Hospital La Paz (PI-643), and all the subjects signed a written informed consent to participate in the study.

Twenty-nine subjects, 15 men and 14 women, with age ranging from 18 to 28 years, volunteered to participate in this study (Table 1). Subjects were moderately active (3–5h·wk^-1^ of exercise with at least one year of experience in strength training). A pre-participation screening including health history and physical examination was performed prior the start of the study. Smokers or individuals reporting a history of diabetes, cardiovascular disease, or metabolic disorders were excluded.

**Table 1:** Subjects characteristics (mean ± SD).

	Men	Women	All
**Age (years)**	22.5 ± 2.6	20.7 ± 3.4*	21.6 ± 3.1
**Body mass (kg)**	76.7 ± 6.4	60.4 ± 5.2*	68.5 ± 10.1
**BMI (kg·m**^**-2**^**)**	24.4 ± 1.9	22.2 ± 1.5*	23.3 ± 2
**Body Fat (%)**	16.1 ± 6.4	27.7 ± 3.8*	21.9 ±7.9
**Body Fat Free (kg)**	61.3 ± 4.9	41.8 ± 3.9*	51.6 ± 10.8
**Bone Mineral Content (kg)**	3.5 ± 0.4	2.6 ± 0.4*	3.0 ± 0.6
**VO**_**2max**_ **(mL·kg**^**-1**^**·min**^**-1**^**)**	57.2 ± 5.9	48.6 ± 5.6*	52.9 ± 7.2

BMI: Body mass index; VO_2max_: Maximum oxygen consumption.

* Differences with men (P<0.001).

A National Strength and Conditioning Association (NSCA) certified personal trainer specialist ensured that all subjects adhered to proper technique during their testing session. The participants visited the laboratory on 14 occasions. During the first visit, VO_2max_ was assessed using an incremental test on a treadmill. Measurements of body composition were performed during the second visit. The resting metabolic rate was evaluated in the third visit, and the next eight visits (fourth to eleventh) were used to calculate the 15 repetition maximum (15 RM). During the last three visits, participants performed in a randomized counterbalanced order three different CWT of the same duration involving: i) exercise performed using machines (CM), ii) free weight exercises (FW) or iii) a combination of free weight exercises and treadmill running (CE).

### Maximal oxygen consumption (${\dot{V}O}_{2\max}$)

The incremental test to measure ${\dot{V}O}_{2\max}$ was performed on a treadmill (H/P/COSMOS 3P ^®^ 4.0, H / P / Cosmos Sports & Medical, Nussdorf-Traunstein, Germany). Volume and composition of expired gasses were measured using an automated system (Jaeger Oxycon Pro, Erich Jaeger, Viasys Healthcare, Germany) [13, 14]. After a 3 min warm-up at 6 km·h^-1^, the speed was increased 0.25 km·h^-1^ every 15 s until volitional exhaustion of the subject. Throughout the test, the treadmill elevation was always maintained at 1%.

### Body composition

Anthropometric measurements included height (stadiometer; Holtain Limited, Crymych, United Kingdom) and body mass (Lafayette Instruments Company, Lafayette, Indiana, USA). Body fat, fat free mass and bone mineral content were measured by whole body dual-energy x-ray absorptiometry DXA (GE Lunar Prodigy; GE Healthcare, Madison, WI).

### Resting metabolic rate

According to previous studies [15], a minimum of 15 min of steady state, determined as <10% fluctuation in VO_2_ and <5% fluctuation in RER, was considered as criteria for valid resting metabolic rate (RMR) These data were used to calculate RMR according to the formula of Weir [16].

### Determination of repetition maximum

The 15 RM represents approximately 61.2% of 1 RM. All the exercises were performed at 70% of 15 RM, that is 42.8% of 1 RM [17]. The 15 RM for each exercise was tested twice on different days and during the previous two weeks before performing the CWT protocols. Four exercises were tested per day, and a maximum of two attempts were performed in the same day. The test started after a 5 minute cycle ergometer warm-up. The test consisted of 3 sets of 15 repetitions (at 50%, 70% and 90% of the estimated 15 RM) performed with 2 min of recovery between them. Following these 3 sets, subjects rested for 5 min, and then a final set of 15 repetitions was carried out at 100% of the estimated 15 RM. If the subject was able to exceed 15 repetitions, a further attempt was performed after 5 min recovery at + 2.5% of the estimated 15 RM. On the contrary, the weight was decreased by 2.5% if the subject did not achieve 15 repetitions [12, 18]. All tests were performed at the same cadence (2 sec concentric: 1 sec eccentric) that would be used later during the CWT protocols. During the CWT tests, all the subjects were verbally encouraged to perform as many repetitions as possible.

The intraclass correlation coefficient of reliability (ICCr) for all exercise was ICCr = 0.995 and ICCr = 0.994 for men and women respectively. The average number of re-tests necessary to assess the actual 15 RM was 1.4 for men and 1.2 for women. All the assessments, data collection sessions and exercises were carried out with the same machines and free weights used during the CWT (Panatta, Italy).

### Circuit weight training

**Fig 1:**
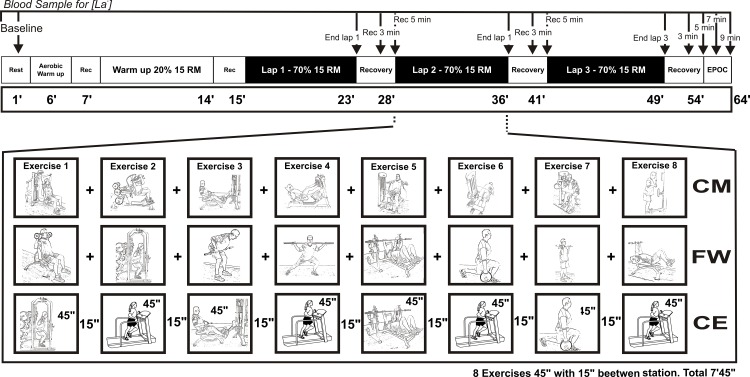
Exercise protocol. Circuit Machine training protocol, Free Weight-training protocol and Combined Exercise training protocol, all with the same duration, intensity, cadence, etc.

Participants came to the laboratory on three non-consecutive days to carry out three different CWT session in a randomized order, at similar times of day, state of nutrition and hydration. Prior to starting the CWT protocol, a 20-G (men) or 18-G (women) catheter was placed and fixed in the antecubital vein. The protocol started with the first lactate measurement at rest prior to exercise. Subsequently, subjects performed a warm-up which involved 5 min running on a treadmill at 50% of the heart rate reserve (HRR) followed by 1 min rest and a first lap (each complete circuit with 8 stations-exercises) to the CWT at 20% of 15 RM. A 1 min recovery was provided before the subjects preformed 3 laps to the corresponding CWT(see Fig 1). Blood lactate was collected after each lap (see below). The protocol finished with 15 min of recovery while the last blood samples were obtained during the excess post-exercise oxygen consumption (EPOC) period.

The Circuit Machine training protocol (CM) was performed as follows:

1) Shoulder press machine, 2) hack squat, 3) seated cable row, 4) leg press, 5) seated chest press, 6) leg curl, 7) biceps curl machine and 8) cable triceps extensions. All the exercises were performed using resistance machines (Panatta, Italy).

The Free Weight training protocol (FW) was performed as follows:

1) dumbbells shoulder press, 2) barbell squats, 3) barbell row, 4) barbell side split squat, 5) bench press, 6) barbell split squat, 7) barbell biceps curl and 8) lying triceps extension.

The Combined Exercise training protocol (CE) was performed as follows:

1) barbell squats, 2) running, 3) seated cable row, 4) running, 5) barbell split squat, 6) running, 7) barbell biceps curl and 8) running.

Each resistance exercise involved 15 repetitions at 70% of 15 RM following a 2 s:1 s cadence for the concentric and eccentric phases respectively (45 s per exercise). The cadence was controlled by sounds recorded on a compact disc. Running during the CE was performed at 70% of the HRR. Between exercises, 15 s allowed for the subjects to change from one station to the next. The total duration of one lap of the circuit was 7 min and 45 s. Since each circuit consisted of 3 laps, the total duration was 23 min and 15 s, and the entire session (including warm-up) took 64 min (Fig 1).

### Energy expenditure estimations

Analysis of the expired gases was performed during the whole protocol using a Jaeger Oxycon Mobile (Erich Jaeger, Viasys Healthcare, Germany) portable metabolic system [19, 20]. Heart rate (HR) was continuously recorded each 15 s using a heart rate monitor (Polar Electro Oy, Kempele, Finland) interfaced with the gas analyzer. All the data [VO_2_, RER, HR and ventilation] are presented as the average of the whole CWT (i.e. 3 laps). Additionally, subjects were asked about their overall feelings and ratings of perceived exertion (RPE) at the end of each CWT, using the Borg CR10 Scale [21].

Blood samples (~2 mL) were drawn in heparinised syringes at baseline and immediately post exercise (End Lap), 3 min (Rec 3 min) and 5 min (Rec 5 min) after the last exercise on each lap and prior to the start of the next lap. Additional blood samples were taken 7 (Rec 7 min) and 9 min (Rec 9 min) after the last lap. Lactate concentration ([La^-^]) was immediately measured using a lactate analyzer (YSI Model 1500 Sport Lactate Analyzer; Yellow Springs Instrument Co, Yellow Springs, Ohio, USA).

Exercise EE for aerobic and anaerobic metabolism was converted as 1 litre of O_2_ = 5.0 kcal. Upon completion of the work period (i. e. after the last lap), subjects immediately rested while standing upright, and EPOC was recorded until it fell below the respective 5-min resting O_2_ uptake measurement (from the RMR test). EPOC was calculated as 1 Liter of O_2_ = 4.64 kcal to exclude rapid glycolytic ATP re-synthesis as part of the conversion of oxygen uptake into EE; in this regard, EPOC represented aerobic energy costs only [12, 22]. Aerobic EE was defined as the difference between the total aerobic EE and the basal standing EE (RMR).

Anaerobic glycolytic EE (i.e., rapid glycolytic ATP re-synthesis) was estimated using recovery peak blood lactate (use of the "anaerobic" ATP/PC stores was assumed to be accounted for as part of EPOC [23]). After the first circuit lap, the Δ[La^-^] was obtained by subtracting resting values from peak [La^-^] reached during recovery. For the next circuit laps, Δ[La^-^] was obtained as the difference of the lowest measurement in the previous recovery and the peak [La^-^] reached during the next recovery period [12]. Measures of Δ[La^-^] were converted to oxygen equivalent values as 3 ml O_2_·kg^-1^ body weight per mmol of Δ[La^-^][12]. Aerobic EE was defined as the difference between the total aerobic EE and the basal standing EE (RMR).

Exercise EE was defined as the aerobic EE added to the anaerobic EE based on the Δ[La^-^] (mean of the three Δ[La^-^] measured during the complete circuit protocol). Total EE was recorded as the sum of aerobic and anaerobic exercise energy expenditures and EPOC [11].

### Statistical analysis

All data are reported as mean ± standard deviation (SD). The independent *t*-test was used to assess differences in body composition and VO_2max_ between genders. The intraclass correlation coefficient was used to assess the reliability in the test-retest measurements. One way analysis of variance (ANOVA) was used to determine differences between CWT protocols (CM, FW, CE) in all studied dependent variables (physiological parameters, EE variables and RPE). Two-way ANOVA (3 protocols x 12 measurements) with repeated measures was used to determine differences between CWT protocols (CW, FW, CE) at each blood [La^-^] measurement. Compound symmetry, or sphericity, was verified by the Mauchly test. When the assumption of sphericity was not met, the significance of *F* ratios was adjusted according to the Greenhouse-Geisser procedure. Multiple comparisons of ANOVAs were made with the Bonferroni *post hoc* test. The dependent *t*-test was used to assess the contribution of anaerobic EE within the CWT. The level of statistical significance was set at α = 0.05 for all analyses.

## Results

The results regarding the physiological response to the three different CWT protocols are presented in Table 2. The average VO_2_, as well as the percentage of VO_2max_, during the entire protocol was significantly higher in CE compared to FW and CM. On the other hand, the lowest values of RER, [La^-^] and [La^-^]_max_ were observed in CW, while the greatest values were measured during CM. These results were consistent when data were analyzed within gender or when men and women were pooled together (Table 2).

**Table 2:** Physiological parameters (mean±SD) measured during Circuit Machine training protocol (CM), Free Weight training protocol (FW) and Combined Exercise training protocol (CE) in men (n = 15) and women (n = 14). Data correspond to the average of the whole circuit weight training (3 laps), except for [La^-^]_max_.

	Men			Women			All		
	CM	FW	CE	CM	FW	CE	CM	FW	CE
**VO**_**2**_ **(mL·kg**^**-1**^**·min**^**-1**^**)**	22.2±2.5	26.0±2.0 ^a^	32.0±3.3 ^a^^b^	18.5±1.9	22.4±2.1^a^	27.7±2.5^a^^b^	20.4±2.9	24.2±2.8 ^a^	29.9±3.6 ^a^^b^
**VO**_**2**_ **(%VO**_**2max**_**)**	38.4±4.6	45.8±4.3 ^a^	56.4±6.9 ^a^^b^	38.6±4.8	46.7±5.8 ^a^	57.7±4.5 ^a^^b^	38.5±4.6	46.2±5.0 ^a^	57.0±5.8 ^a^^b^
**RER**	1.09±0.05	1.00±0.03 ^a^	0.97±0.04 ^a^	1.01±0.06	0.94±0.05 ^a^	0.91±0.04 ^a^^b^	1.05±0.07	0.97±0.05 ^a^	0.94±0.05 ^a^^b^
**Ventilation (L·min**^**-1**^**)**	69.1±10.5	75.4±12.4 ^a^	79.7±8.6 ^a^	44.0±6.6	48.1±7.2	53.3±8.5 ^a^	57.0±15.4	62.2±17.2	67.0±15.9
**HR (bpm)**	152±11	160±11^a^	160±10 ^a^	141±17	150±14 ^a^	155±11 ^a^	147±15	155±14 ^a^	158±10 ^a^
**[La**^**-**^**] (mmol·L**^**-1**^**)**	10.6±1.9	9.8±1.5	6.2±1.8 ^a^^b^	7.5±2.2	6.1±1.7^a^	3.9±1.1 ^a^^b^	9.1±2.5	8.0±2.5^a^	5.1±1.9 ^a^^b^
**[La**^**-**^**]**_**max**_ **(mmol·L**^**-1**^**)**	12.8±2.2	11.7±2.3	6.9±2.3 ^a^^b^	8.5±2.5	6.9±1.9 ^a^	4.5±1.4 ^a^^b^	10.7±3.2	9.4±3.2	5.8±2.3^a^^b^
**RPE**	8.9±0.2	9.5±0.2 ^a^	7.6±0.3 ^a^^b^	8.3±0.2	8.4±0.2	7.6±0.3 ^a^^b^	8.4±0.2	9.0±0.2 ^a^	7.6±0.3 ^a^^b^

${\dot{V}O}_{2}$: Oxygen uptake; RER: Respiratory exchange ratio; HR: Heart rate; [La^-^]: Lactate concentration; [La^-^]_max_: maximal lactate concentration during training protocols; RPE: rating of perceived exertion.

^a^ p<0.05 with CM

^b^ p<0.05 with FW.

**Fig 2:**
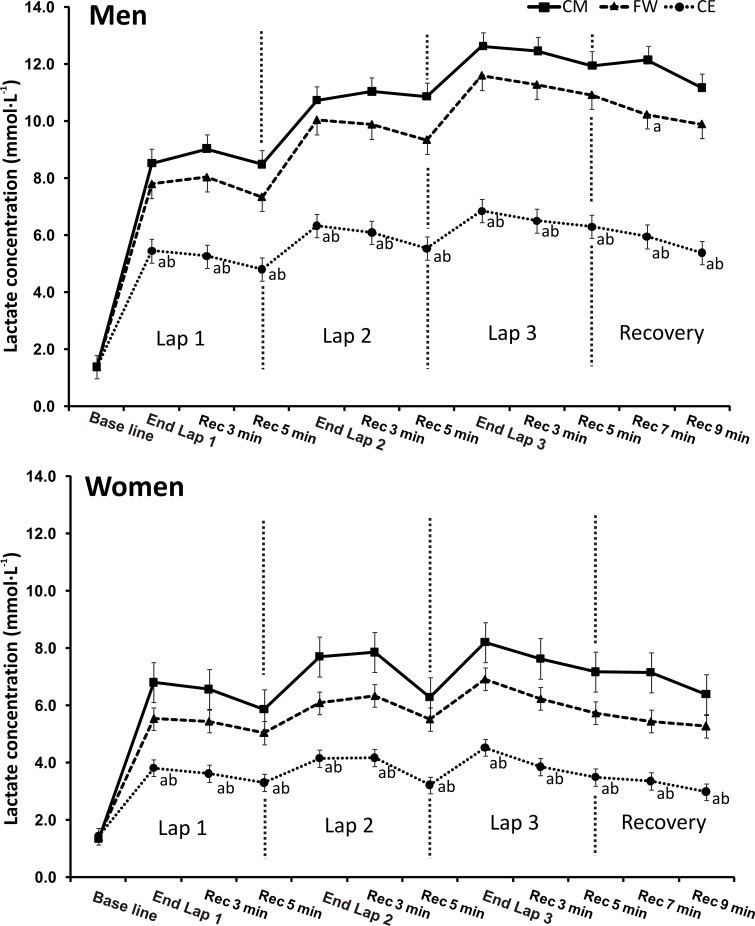
Blood lactate concentration measurements (mmol·L^-1^) during training protocols. CM: Circuit Machine training protocol; FW: Free Weight-training protocol; CE: Combined Exercise training protocol. Data are reported as mean ± SEM for women (n = 14) and men (n = 15). ^a^ p<0.05 with CM. ^b^ p<0.05 with FW.

Further analysis of lactate kinetics revealed that, in both men and women, [La^-^] was systematically higher in CM and FW vs. CE; significantly lower values were found in CE vs. CM and FW (Fig 2). A lower [La^-^] in CE was accompanied by a significantly lower RPE, while the FW was perceived as the most difficult CWT protocol (Table 2).

In men and women, the greatest aerobic EE, as well as the aerobic contribution to total EE, was observed when resistance and aerobic exercises were combined (i.e. CE protocol) (Table 3). When the total EE was calculated as the sum of aerobic and anaerobic EE, the CE protocol also produced the greatest values independently of the units in which it was expressed (Table 3).

**Table 3:** Energy expenditure variables in each training protocol (mean±SD) measured during Circuit Machine training protocol (CM), Free Weight training protocol (FW) and Combined Exercise training protocol (CE) in men (n = 15) and women (n = 14).

	Men			Women			All		
	CM	FW	CE	CM	FW	CE	CM	FW	CE
**Aerobic VO**_**2**_ **(mL·kg**^**-1**^**·min**^**-1**^**)**	21.9±3.2	25.8±3.5 ^a^	33.2±3.9 ^a^^b^	17±2.5	20.6±3.2 ^a^	27.6±3.2 ^a^^b^	19.5±3.8	23.3±4.3 ^a^	30.5±4.5 ^a^^b^
**Total VO**_**2**_ **(mL·kg**^**-1**^**·min**^**-1**^**)**	23.6±3.2*	27.3±3.7 ^a^*	34.1±3.8 ^a^^b^*	18.1±2.8*	21.4±3.5 ^a^*	28.1±3.1 ^a^^b^*	20.9±4.0*	24.4±4.6 ^a^*	31.2±4.6 ^a^^b^*
**Aerobic EE (Kcal·min**^**-1**^**)**	8.3±1.0	9.8±1.5 ^a^	12.6±1.6 ^a^^b^	5.1±0.7	6.2±1.0 ^a^	8.3±1.3 ^a^^b^	6.7±1.8	8.0±2.2	10.5±2.6 ^a^^b^
**Total EE (Kcal·min**^**-1**^**)**	8.9±1.0**	10.4±1.6 ^a^**	13.0±1.6 ^a^^b^**	5.4±0.8**	6.4±1.0**	8.4±1.3 ^a^^b^	7.2±2.0**	8.5±2.4 ^a^**	10.8±2.7 ^a^^b^**
**Total EE (Kcal)**	213±24	249±37 ^a^	311±38 ^a^^b^	130±19	154±25	203±31 ^a^^b^	173±48	203±58 ^a^	259±65 ^a^^b^
**Total EE (MET's)**	6.7±0.9	7.8±1.0 ^a^	9.8±1.1 ^a^^b^	5.2±0.8	6.1±1.0 ^a^	8.0±0.9 ^a^^b^	6.0±1.2	7.0±1.3 ^a^	8.9±1.3 ^a^^b^

^a^ p<0.05 with CM

^b^ p<0.05 with FW

* p<0.001 with aerobic EE within the CWT protocol

** p<0.05 with aerobic VO_2_ within the CWT protocol.

**Fig 3:**
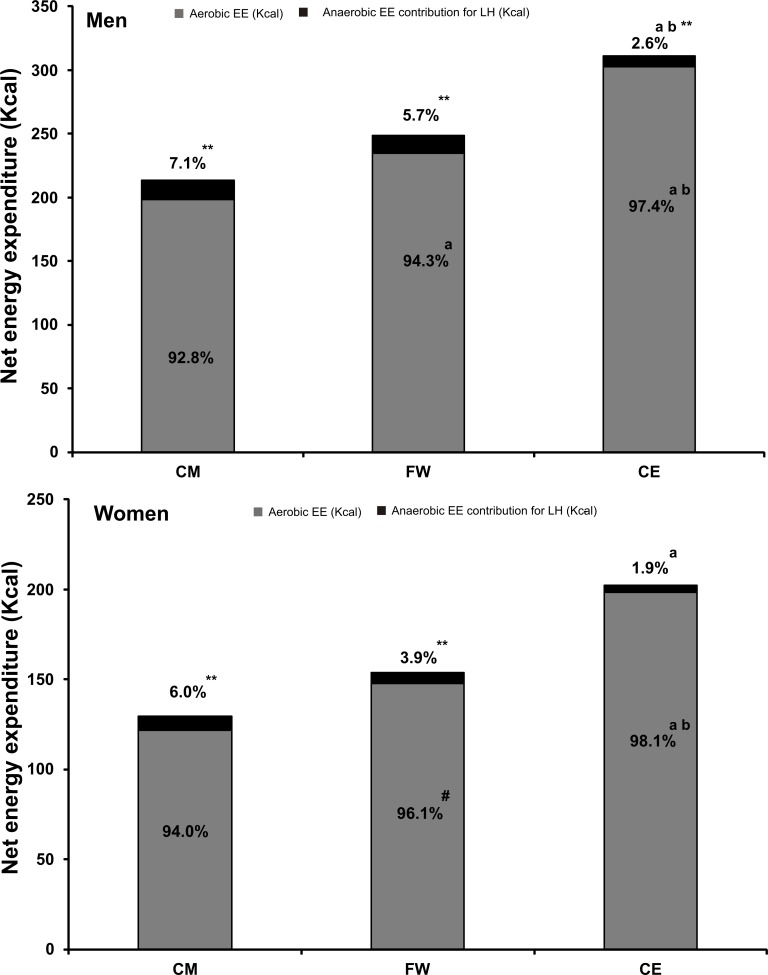
Net energy expenditure (Kcal) in men (n = 15) and women (n = 14) during the entire circuit weight training protocol. Percentages represent the individual contribution of aerobic energy expenditure (grey) and anaerobic energy expenditure (black) to total energy expenditure. CM: Circuit Machine training protocol; FW: Free Weight-training protocol; CE: Combined Exercise training protocol. ^a^ p<0.05 with CM, ^b^ p<0.05 with FW, ** p<0.001 aerobic energy expenditure with total energy expenditure (aerobic + anaerobic) (P<0.001), ^#^ p = 0.06 with CM.

Fig 3 depicts the aerobic and anaerobic contributions to total EE. Aerobic contributions were higher in CE, followed by FW and CM in men and women; the greatest anaerobic contribution was observed during CM followed by FW and CE. In addition, when the anaerobic contribution to total EE was taken into account, total EE was significantly higher than aerobic EE in all three CWT protocols and in men and women, respectively (Table 3, Fig 3).

## Discussion

The main finding of this study is that aerobic exercise interposed in a circuit resistance exercise increases oxygen consumption and energy expenditure more than conventional circuit training, in addition to lowering perceived exertion.

The lack of an estimate of anaerobic EE may underestimate total EE during isolated resistance exercises [8, 11, 12]. To the best of our knowledge, this is the first study attempting to estimate both the aerobic and anaerobic contributions to total EE during the entire CWT protocols, a type of exercise considered by some to be aerobic.

The physiological responses to CWT depend on a number of variables such as rest intervals between exercises [24], intensity [25, 26], gender [26–29], and the combination of endurance and resistance exercises in the same circuit [27]. We used submaximal intensity (70% of 15RM), short rest intervals between exercises (15 seconds), and 1:2 (concentric: eccentric) frequency in an attempt to reproduce an aerobic-type format. Our data show that the CWT with both resistance and endurance (running) exercises produced the highest VO_2_ and the lowest RPE and [La^-^]; this was also the only CWT protocol producing a VO_2_ higher than 50% VO_2max_, the minimum intensity recommended by the American College of Sport Medicine to achieve exercise-induced cardiovascular adaptation. We further investigated total EE and the contributions of both aerobic and anaerobic EE. EE during CWT has been previously investigated using metabolic carts [26] or accelerometers [30–32], where aerobic-only estimates of EE are taken into account. The aerobic methods used to calculate the EE of anaerobic activities may significantly affect EE estimation [7]. Scott et al. have estimated total EE during resistance exercises using isolated exercises, demonstrating that anaerobic contributions to total EE during bench press performed at 37%, 46% and 56% 1RM was 46%, 51% and 50%, respectively [11]. To apply the same methodology through an entire CWT, we carefully evaluated the dynamics of lactate accumulation during three CWT protocols, taking into account the anaerobic contribution to total EE[33]. Our results show a modest though significant contribution of anaerobic EE (between 1.9% and 7.1%, see Table 3 and Fig 2) [33]. We further suggest that increasing the number of repetitions and reducing the intensity (i.e. increasing mechanical work) reduces the anaerobic contribution to total EE. Our results demonstrate that even small contributions of anaerobic EE, if not taken into account, lead to an underestimation of total EE during CWT independently of the selected exercises (machines, free weight, or combination of resistance exercise with running).

Finally, we also found differences between those protocols involving resistance exercises only: the FW protocol elicited a greater total EE and aerobic EE. These differences may be explained by the participation of muscles acting as stabilizers during free-weight exercises [34]. The CWT protocol combining free-weight exercises and running (CE protocol) produced the highest EE with the lowest anaerobic EE contribution. Since concurrent training has a synergistic effect on cardiovascular and strength measures [35] when certain factors are controlled for, our CE protocol, which produced the highest EE with the lower RPE, may prove useful in interventions using subjects who choose to exercise at lower intensities. In fact, a recent study has demonstrated the beneficial effects of the inclusion of resistance exercises into an aerobic training program in overweight adults [6].

The most frequent effect of resistance training inclusion in a weight loss program is the maintenance or increase of the muscle mass [36]. This inclusion of strength training influences also post-exercise energy expenditure, given its relation with the raise in basal metabolic rate induced by muscle increase [37]. Furthermore, the increase in fat free mass is a powerful predictor for weight regain after the intervention, as the subjects who included resistance training in their programs and were more adherent to it regain less weight one years after the intervention [38, 39]. In this respect, we must highlight the lower perceived exertion of the CE protocol, which could increase the adherence to the training programme without eliminating the benefits of strength training.

From a practical point of view, the present study points out the importance of anaerobic contribution to total EE during CWT. Reducing the intensity but increasing the number of repetitions (i.e. increasing mechanical work) enhances EE, and consequently the idea of “the higher the weight lifted, the higher the EE” is not applicable [40, 41]. In addition, a combination of resistance exercises and running produces VO_2_ above 50% VO_2max_, the highest EE, and the lowest perception of effort. This may be beneficial for those subjects who do not like traditional strength training or continuous aerobic training. Also, this finding can be applied to overweight and obese patients who wish to increase their energy expenditure to lose weight.

The present study provides better understanding on how to design programs with different types of exercise that are equivalent in energy expenditure, allowing to accurately compare the effect of these exercise on health outcomes.

Combined exercise induced greater oxygen consumption and energy expenditure than conventional circuit training.

Anaerobic EE ranges from 1.9% to 7.1% during circuit weight training. Despite this modest contribution to total energy expenditure, lack of quantification of the anaerobic contribution of a given exercise produces a systematic underestimation of total energy expenditure independent of the exercises included in a circuit weight training protocol. Although the implications of this underestimation in the calculation of energy balance during longer interventions needs to be further investigated, anaerobic energy expenditure should be considered in future studies that utilize any form or intensity of resistance training.

## Practical Implications

1. Based on the findings of the present study, alternating cardiovascular exercise with resistance exercise in CWT programs can significantly increase energy expenditure with the potential to promote weight loss at a lower perceived exertion.
2. This alternative type of exercise could motivate those who do not like traditional strength training or continuous cardiovascular training.
3. Conventional energy expenditure tables containing strength training methods underestimate the energy cost of these physical activities, since the anaerobic contribution has not been usually taken into account.
